# Addressing the role of PKD3 in the T cell compartment with knockout mice

**DOI:** 10.1186/s12964-022-00864-w

**Published:** 2022-04-19

**Authors:** Jiří Koutník, Verena Neururer, Thomas Gruber, Sebastian Peer, Natascha Hermann-Kleiter, William J. Olson, Verena Labi, Michael Leitges, Gottfried Baier, Kerstin Siegmund

**Affiliations:** 1 Institute of Cell Genetics, Medical University Innsbruck, Innsbruck, Austria; 2 Present Address: Apoptosis, Cancer, and Development Laboratory, Equipe labellisée ‘La Ligue’, LabEx DEVweCAN, Centre de Recherche en Cancérologie de Lyon, INSERM U1052-CNRS UMR5286, Centre Léon Bérard, Université de Lyon, Université Claude Bernard Lyon1, 69008 Lyon, France; 3 Present Address: Institute for Biomedical Aging Research, University of Innsbruck, Innsbruck, Austria; 4 Institute of Developmental Immunology, Medical University Innsbruck, Innrain 80-82, 6020 Innsbruck, Austria; 5 Division of BioMedical Sciences, Faculty of Medicine, Craig L Dobbin Genetics Research Centre, Memorial University of Newfoundland Health Science Centre, 300 Prince Philip Drive, St. John’s, NF A1B 3V6 Canada

**Keywords:** T lymphocyte, TCR signaling and activation, Protein kinase D, PKD

## Abstract

**Background:**

The Protein kinase D3 (PKD3) has been implicated in signal transduction downstream of the T cell receptor (TCR). However, its role for the activation of primary T lymphocytes has not been elucidated so far.

**Methods:**

Expression of PKD isoforms in primary murine T cells was determined by RT-PCR and SDS-Page. A germline PKD3-knockout mouse line was analyzed for its immune response to OVA/alum intraperitoneal immunization. Phenotyping of the T cell compartment ex vivo as well as upon stimulation in vitro was performed by flow cytometry. Additionally, cytokine expression was assessed by flow cytometry, RT-PCR and Luminex technology.

**Results:**

PKD expression in T cells is modulated by TCR stimulation, leading to a rapid down-regulation on mRNA and on protein level. PKD3-deficient mice respond to immunization with enhanced T follicular helper cell generation. Furthermore, peripheral PKD3-deficient CD4^+^ T cells express more interleukin-2 than wild type CD4^+^ T cells upon TCR stimulation ex vivo. However, purified naïve CD4^+^ T cells do not differ in their phenotype upon differentiation in vitro from wild type T cells. Moreover, we observed a shift towards an effector/memory phenotype of splenic T cells at steady state, which might explain the contradictory results obtained with pan-T cells ex vivo and naïve-sorted T cells.

**Conclusion:**

While PKD3-deficiency in vivo in mice leads to a skewing of the T cell compartment towards a more activated phenotype, this kinase seems to be dispensable for naïve CD4^+^ T cell differentiation in vitro.

**Video Abstract**

**Supplementary Information:**

The online version contains supplementary material available at 10.1186/s12964-022-00864-w.

## Background

T cell receptor (TCR) signaling initiates T cell activation as well as differentiation and is thus critical for proper effector T cell responses during an immune reaction. Briefly, (antigen-specific) activation of T cells via engagement of the TCR complex by peptide-loaded MHCs (major histocompatibility complex) initiates a signaling cascade involving several protein kinases. Early upon stimulation, lymphocyte-specific protein tyrosine kinase (LCK) phosphorylates immunoreceptor tyrosine-based activation motifs (ITAMs) in the cytoplasmic domain of the CD3ζ chain leading to the subsequent recruitment of ζ-chain-associated protein (ZAP-70), which in turn becomes activated by phosphorylation and further propagates the signal. Subsequently, effector signaling molecules localize to the plasma membrane. So does for example phospholipase Cγ1 (PLCγ1), which catalyzes the formation of the second messengers diacylglycerol (DAG) and inositol-(1,4,5)-trisphosphate (InsP3) upon activation. DAG in turn binds and activates among others members of the classical and novel protein kinase C (PKC) family. It is well established that, in particular, the classical PKC isoform PKCα, and the novel PKC isoform PKCθ are critical for T cell function and play a decisive role in the nature of effector responses [1–6]. Thus, PKCθ-deficient T cells show impaired interleukine (IL)-2 expression and proliferation.

Other DAG-regulated serine/threonine kinases are members of the PKD family. Although initially described as PKC-family members, later they have been classified within the Ca^2+^/calmodulin-dependent kinase (CaMK) superfamily based on the homology of their kinase domain. Of note, classical as well as novel PKCs are implicated in the phosphorylation events that contribute to the activation of PKDs [7, 8]. Hence, the activation of PKDs seems to be linked to TCR signaling. Interestingly, Enrique Rozengurt has suggested that “*it seems that a variety of biological responses attributed originally to PKCs are in fact executed by PKD*” [9]. In mammals, the PKD family comprises three highly homologous isoforms PKD1, 2 and 3, encoded by three different genes (*prkd1-3*). Notably, members of the PKD family have been implicated in the regulation of various biological processes in different cell types, including cell proliferation and differentiation, cell migration, adhesion and polarization as well as vesicle trafficking and protein secretion (reviewed in 9). Naturally, their function has also been linked to several pathologies such as cardiac hypertrophy, cancer and hyperalgesia (for reviews, see [10–12]). Moreover, PKDs are involved in regulation of both, innate and adaptive immune responses. Nevertheless, the specific tasks of the distinct PKD isoforms on a molecular level in T cell subsets remain elusive. One reason for this is that for long time it was impossible to distinguish between the highly homologous PKD1 and PKD2 proteins using antibodies [13]. Moreover, some studies require critical reviewing, since they either do not specify the analyzed isoform or were performed with ectopically expressed PKD1, which later turned out not to be present in T lymphocytes [13, 14]. In fact, Irie et al*.* and Matthews et al*.* showed that T lymphocytes express only two PKD isoforms: predominantly PKD2 and to a lesser extent PKD3.

In mature T cells several studies have stressed the importance of PKD2 for proper T cell function. When lacking PKD2, T cells show an impaired antigen receptor-induced cytokine production and T cell-dependent antibody response [13] as well as a defect in multi-vesicular body (MVB) formation, resulting in defects in cytotoxic activity [15]. Navarro et al*.* have concluded, that PKD2 acts as a digital amplifier upon TCR stimulation [16] and recently, PKD2 was implicated as a negative regulator of follicular T helper cell (Tfh) development [17]. Thus, while the role of PKD2 in T lymphocytes has become more clear in recent years, hitherto, little is known about PKD3’s distinctive functions in T lymphocytes. Conditional deletion of PKD2 and PKD3 impaired thymocyte development, whereas individual deletion had no effect [18]; suggesting a redundant function of these two PKD isoforms.

We tackle this gap by focusing on PKD3’s role in the T cell compartment. To this end, we have characterized T cell-dependent immune responses of a PKD3-deficient (PKD3^−/−^) mouse line upon immunization in vivo as well as T cell activation after stimulation in vitro. Briefly, we have observed a shift in the T cell compartment towards an effector/memory phenotype, which resulted in enhanced immune response in vivo as well as a stronger activation response in vitro.

## Material and methods

### Mice

PKD3-deficient mice were generated by homologous recombination in ES cells deleting exon 2 and 3 using the Cre-lox system ([19]; details of the knockout strategy are part of a manuscript by the Leitges´ laboratory; currently under revision). For standard genotyping, genomic DNA was prepared from ear tag biopsies and used as the template in a specific PCR using the following primers: WT_fwd_: GCAGTGGCTGATCATGTATTGAGCAG; WT_rev_: CTGACAGGACAACTTCTACCAGGTC; Del_fwd_: GCCACACTGTACCCCAGCTCATG; Del_rev_: GGGTAGAGCGCTCTTCACAGAG (all custom synthesized from Eurofins Genomics Germany GmbH) and the following thermocycler protocol: 40 cycles of 95 °C for 30 s, 61 °C for 30 s and 72 °C for 45 s. The WT-primer pair gave rise to a 488-bp wild type fragment, which only occurs when no deletion is present, whereas the Del-primer pair generate a deletion specific 277-bp fragment, which was used to identify the PKD3^−/+^ allele. The mice were backcrossed to C57BL6/N. The PKD3^−/+^ heterozygous breeding pairs were housed under specific-pathogen-free conditions at the animal facility of the Medical University of Innsbruck. All animal experiments were conducted in accordance with the Austrian Animal Welfare Law and Animal Experimental Act, and were approved by the Committee of the Animal Care of the Austrian Federal Ministry of Science and Research (GZ: 2020-0.345.522). For experiments, 10 to 16 weeks old male and female mice were used.

### Splenocyte isolation, T cell sorting and in vitro stimulation

Single-cell suspensions of splenocytes were prepared by mechanical disintegration using metal sieves or 100 µM cell strainers (Falcon). Thereafter, erythrocytes were removed by lysis (0.15 M NH_4_Cl, 10 mM KHCO_3_, 0.1 mM EDTA) for 2–6 min at RT, followed by a wash/filter step using PBS, 0.5% BSA, 2 mM EDTA or RPMI-1640 (PAN Biotech) and 40 µM cell strainers (Falcon). Viable cell counts were determined after staining with AO/PI Cell Viability Kit (F23001-LG, Biocat) using a LUNA Automated Cell Counter (Logos Biosystems).

All T cell subsets were separated by negative selection using the following MACS technology-based isolation kits: 130-095-130 (pan-T cells), 130-090-860 (CD4^+^ T cells), 130-104-453 (naïve CD4^+^ T cells); along with pre-separation filters, LS columns and a QuadroMACS separator (all Miltenyi Biotec) according to the manufacturer’s instructions. Separation purity was checked by flow cytometry.

Purified T cells were cultured in RPMI-1640 medium (PAN Biotech) supplemented with 10% heat-inactivated FCS (Biowest), 100 U/ml penicillin, 100 µg/ml streptomycin, 2 mM l-glutamine (all PAN Biotech) in a humidified incubator at 37 °C and 5% CO_2_.

For phospho-immunoblot analyses, sorted CD4^+^ T cell were rested for 2 h in X-Vivo 20 medium (BE04-448Q from Lonza) at a cell density of 1 × 10^7^/ml in a 1.5 ml conical tube in a humidified incubator at 37 °C and 5% CO_2_. Thereafter, equal volume of X-Vivo 20 medium containing 10 µg/ml anti-CD3ε (clone 145-2C11; BioXcell), 5 µg/ml anti-CD28 (clone 37.51, BioXCell) and 5 µg/ml mouse anti-Armenian and Syrian Hamster IgG1 (550637, BD Biosciences) were added. After 5, 15 or 30 min the cells were pelleted by a quick centrifugation at maximal speed and lysed for further analysis by SDS-Page (for details see below). Alternatively, cells were stimulated by 50 ng/ml phorbol-12,13-dibutyrate (PDBu, mimicking DAG binding) and 500 ng/ml ionomycin (Ca^2+^ ionophore) (P1269 and I0634, both from Sigma).

For in vitro polyclonal stimulation, T cells were seeded into a flat-bottom plate (4.5 × 10^5^/200 µl in a 96-well plate) coated with 5 µg/ml anti-CD3ε. Additionally, anti-CD28 antibodies were added at a final concentration of 1 µg/ml to the supplemented RPMI-1640 culture medium. To address (intracellular) cytokine expression by flow cytometry, Golgi Plug (1:1000; 555029, BD) was included during the 4 h stimulation with PDBu/ionomycin.

To inhibit PKDs in T cells, one of two low molecular weight inhibitors (CRT0066101 or CID2011756, both from Tocris) was added to the cell culture one hour prior to stimulation with anti-CD3/CD28 antibodies or PDBu/ionomycin (as described above).

For differentiation towards a Th1-phenotype, sorted naïve CD44^−^CD4^+^ cells were stimulated with anti-CD3/CD28 antibodies (as described above) in supplemented RPMI-1640 culture medium additionally supplemented with recombinant murine (rm) IL-12(p70) (10 ng/ml; 577004 from Biolegend), rmIFN-γ (20 ng/ml; 14-8311-63 from eBiosciences) and anti-IL-4 (5 µg/ml; 14-7041-85). Th0 phenotype was obtained by polyclonal stimulation in the presence of anti-IL-4, anti-IL-12 (14-7122-85) and anti-IFN-γ (14-7311-85) all at 5 µg/ml, from eBiosciences.

### SDS-Page and immunoblotting

Cells were lysed on ice for 20 min (frequently vortexed) in the following buffer: 5 mM Na_3_VO_4_, 5 mM NaP_2_P, 5 mM NaF, 5 mM EDTA, 150 mM NaCl, 50 mM Tris (pH = 7.3), 2% NP-40, 50 µg/mL aprotinin and leupeptin. Subsequently, the lysed cells were centrifuged for 15 min at 15,000 g and the supernatant containing cellular proteins was mixed with SDS-loading buffer (Thermo Fisher Scientific) and incubated for 5 min at 95 °C. SDS-PAGE was performed using a 4–12% Bis–Tris gel (Thermo Fisher Scientific) in a MOPS-based buffer (50 mM MOPS, 50 mM Tris, 3.5 mM SDS, 1 M EDTA; pH = 7.7). Afterwards, proteins were transferred to a PVDF membrane (Immobilon-P, Merck) using the semi-dry method. Unspecific binding was blocked with 5% non-fat milk powder (for PKD2) or 5% BSA (for PKD3 and phospho-PKD) in TBS-T (5 mM Tris, 75 mM NaCl, 0.5% v/v Tween). Membranes were incubated overnight at 4 °C with primary antibodies specific to β-actin (C-11, sc-1615, 1:2000), PKD2 (F-2; sc-374344, 1:1000), both from Santa Cruz Biotechnology, PKD3/PKCν (D57E6) Rabbit mAb #5655 and Phospho-PKD/PKCμ (Ser744/748) Antibody #2054, both from Cell Signaling, 1:1000, sealed in plastic shields. Subsequently, after washing with TBS-T the membrane was incubated for one hour at RT with horseradish peroxidase-coupled secondary antibodies (goat anti-mouse, goat anti-rabbit or rabbit anti-goat; all from Pierce). For detection, SuperSignal West Pico Chemiluminescent Substrate (Thermo Scientific) was applied and the bands were visualized using hyperfilm (Amersham Biosciences) and Superfix Enviro Safe 25 together with Roentoroll Enviro Safe AC (Tetenal).

### RNA extraction, cDNA synthesis and quantitative RT-PCR

For gene expression analysis, RNA was isolated with the RNeasy Mini Kit (including on column DNAse digest) and transcribed into cDNA using the Omniscript RT Kit (205113, Qiagen) with oligo (dt) 15 primer and random primers (C110A and C118A, both Promega) according to manufacturers’ instructions. Recombinant RNasin ribonuclease inhibitors (Promega) were included during cDNA synthesis. Quantitative real-time PCR was performed based on the TaqMan technology (7500 Fast Real-Time PCR instrument; Applied Biosystems). For this, Luna Universal Probe qPCR Master Mix (M3004E from NEB) and respective TaqMan gene expression assays from Applied Biosystems were used: Mm00626821_m1 (prkd2), Mm00472455_m1 (prdk3) and mouse GAPDH endogenous control (catalog nr. 4352339E). PCR program: initial denaturation at 95 °C for 60 s, then repetitive cycles of 15 s at 95 °C and 30 s at 60 °C. All measurements were performed in duplicates. Data were quantified using a comparative Ct method. Samples were normalized to the housekeeping gene (GAPDH) and to an ex vivo condition (2^ΔΔCt^ method).

### Flow cytometry and luminex technology

Flow cytometric analyses were performed on FACS Canto II (4-2-2 configuration, BD Biosciences) and subsequent data processing using the FlowJo software. For surface staining, cells were incubated for 5 min with FcR block (anti-CD16/32; BD Biosciences) prior to addition of the antibody mix in PBS, 0.5% BSA, 2 mM EDTA. Subsequently, the cells were incubated for 20 min with the antibody solution at 4 °C. Lastly, cells were washed with PBS, 0.5% BSA, 2 mM EDTA and transferred into FACS tubes (all steps at 4 °C). The following antibodies were used for surface stainings: CD3 Pacific blue (clone 17A2; Biolegend), CD3 PE (clone 145-2C11; eBioscience), CD4 V500 (clone RM4-5; BD Biosciences), CD8 Pacific blue (clone 53-6.7; BD Biosciences), CD8 PerCPCy5.5 (clone 53-6.7; Biolegend), CD25 APC (clone PC61.5; eBioscience), CD44 FITC (clone IM7, BD Biosciences), CD45 V500 (clone 30-F11; BD Biosciences), CD62L APC-Cy7 (clone MEL14; Invitrogen), CD127 APC (clone SB/199; Biolegend), CXCR5-bio (clone 2G8; BD Biosciences) and SA-APC-Cy7, PD-1 FITC (clone J43; eBiosciences), GL7 FITC (clone GL7; BD Biosciences), Fas Alexa Fluor 647 (clone Jo2; BD Biosciences), B220 APC-Cy7 (clone RA3-6B2; Biolegend), KLRG1 PE-Cy7 (clone 2F1; Biolegend). When a biotinylated primary antibody was used, the cells were washed once after the initial incubation with the antibody mix and thereafter incubated with streptavidin-conjugates for 15 min at 4 °C; followed by a washing step.

To exclude dead cells from the analysis, the cells were stained with the Fixable Viability Stain 780 (BD Biosciences, diluted 1:2000 in HBSS) for 10 min at RT in the dark according to the manufacturer’s instructions.

To stain intracellular antigens, the cells were first stained for surface antigens, then fixed and subsequently permeabilized. For Ki-67 nuclear staining, the FoxP3 transcription factor staining buffer set (00-5523-00 from eBiosciences) was used. The Ki-67-PE antibody was purchased from BD Biosciences (556027). For staining of cytokines, fixation buffer (420801, Biolegend) and intracellular staining permeabilization wash buffer from Biolegend (421002) were used. The IL-2 APC antibody (clone JES6-5H4) was purchased from BD Biosciences.

For determination of secreted IL-2 or IFN-γ in the cell culture supernatant, we used Luminex xMAP Technology with the respective kits (No. 171G5003M for IL-2 and No. 171G5017M for IFN-γ) according to the manufacturer’s instructions on a Bio-Plex suspension array system (all Bio-Rad).

### Statistical analysis

All data are shown as individual data points with the mean from at least two independent experiments. The number of biological samples (n) is mentioned in each figure description. If not stated otherwise, statistical significance was tested using an unpaired t-test. The analyses were performed using GraphPad Prism Software. A p value < 0.05 was considered statistically significant. For multiple comparisons within one graph, the threshold for statistical significance was adjusted according to the Bonferroni-Holm method. Symbols used in the figures are: *p ≤ 0.05, **p ≤ 0.01 and ***p ≤ 0.001.

## Results

### PKDs are regulated upon T cell activation in vitro

**Fig. 1:**
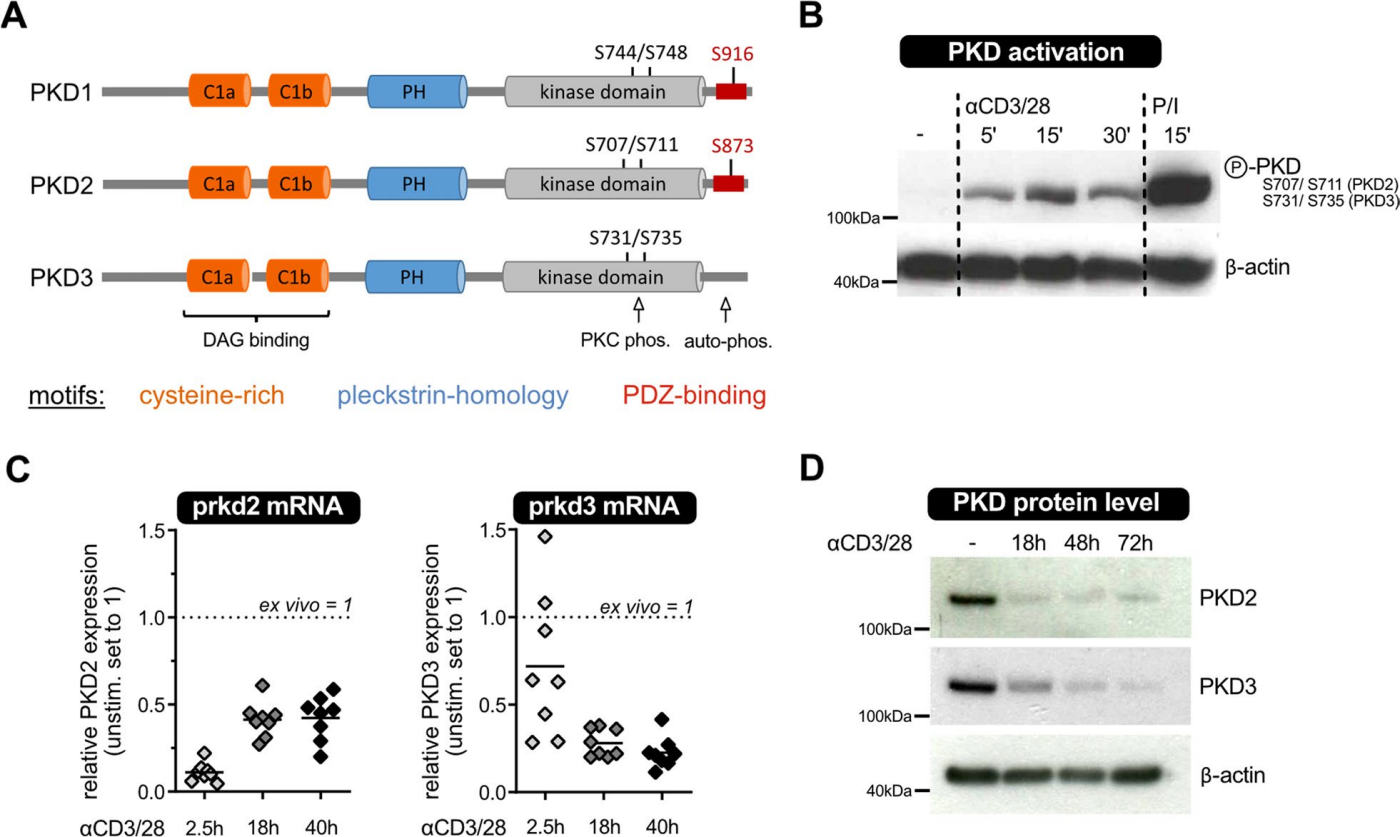
PKD expression in CD4^+^ T lymphocytes. **a** Schematic depiction of the three PKD isoforms showing their conserved domains and phosphorylation sites. **b** Ex vivo MACS-sorted CD4^+^ T cells were stimulated for the depicted period of time with anti-CD3/CD28 antibodies, cross-linked by anti-hamster IgG, or P/I. Activating phosphorylation of PKDs’ serines in their activation loop was analyzed by SDS-PAGE and immunoblot. Actin was used as loading control. **c** PKD2 and PKD3 mRNA expression of CD4^+^ T cells isolated from wild type mice was analyzed after 2.5, 18 and 40 h of stimulation with platebound anti-CD3 and soluble anti-CD28 antibodies by qRT-PCR. The house keeping gene gapdh was used for normalization, and the results are depicted relative to unstimulated T cells ex vivo (set to one; highlighted by dashed line). **d** PKD2 and PKD3 protein expression of MACS-sorted wild type CD4^+^ T cells was analyzed upon in vitro stimulation for 18, 48 and 72 h by SDS-PAGE and immunoblot. Actin was used as loading control

It is now clear that T cells express only two of the three PKD isoform: PKD2 and PKD3 [13, 14]. While the conserved phosphorylation sites within the catalytic domain is present in all three PKD family members, PKD3 is unique with regard to the lacking C-terminal auto-phosphorylation site (Fig. 1A). Thus, PKD3 is considered to be “the most distinct member of the PKD family” [8]. Nevertheless, the activation of all three PKDs is regulated by phosphorylation events.

Thus, analysis of phosphorylation of a pair of serine residues located in the kinase domain/activation loop, which are described as target sites of PKCs, can be used to determine activation status of these kinases [13, 14, 16]. Here, to address whether PKDs are activated in mature T cells upon stimulation, we performed short-term stimulation experiments of primary murine T cells by cross-linking the TCR complex with anti-CD3 and anti-CD28 antibodies. In addition, phorbolester and ionomycin treatment, by-passing the TCR, was included as a positive control [7]. After 5, 15 and 30 min the cells were lysed and phosphorylation was analyzed by SDS-Page and immunoblotting. This treatment rapidly induced phosphorylation of the relevant serine pair in the activation loop of PKDs (Fig. 1B); visible by a strong phospho-specific signal after 5 min of activation.

However, this phospho-specific antibody does not distinguish between PKD isoforms since this phosphorylation site is conserved between all PKD family members (Fig. 1A). Therefore, we wondered whether the expression of the two T cell-expressed isoforms, PKD2 and PKD3, is regulated upon TCR-trigger and if there is any difference between these isoforms. Applying quantitative RT-PCR, we observed downregulation of PKD2 and PKD3 mRNA levels in vitro upon anti-CD3/CD28-treatment of sorted CD4^+^ T cells as well as CD8^+^ T cells (Fig. 1C and data not shown). PKD2 and PKD3 seemed to differ in their mRNA regulation; with a faster downregulation of PKD2 mRNA level compared to PKD3 mRNA, reaching on average 11% of the initial PKD2 amount (versus 72% for PKD3) 2.5 h after cross-linking by anti-CD3 and anti-CD28 antibodies. The PKD3 mRNA expression was reduced to 22% after 40 h of stimulation. Additionally, downregulation of PKD2 and PKD3 expression upon TCR-stimulation in vitro was also observed on protein level by immunoblotting with isoform-specific antibodies (Fig. 1D).

### Enhanced adaptive immune response of PKD3-deficient mice to immunization with OVA/alum

**Fig. 2:**
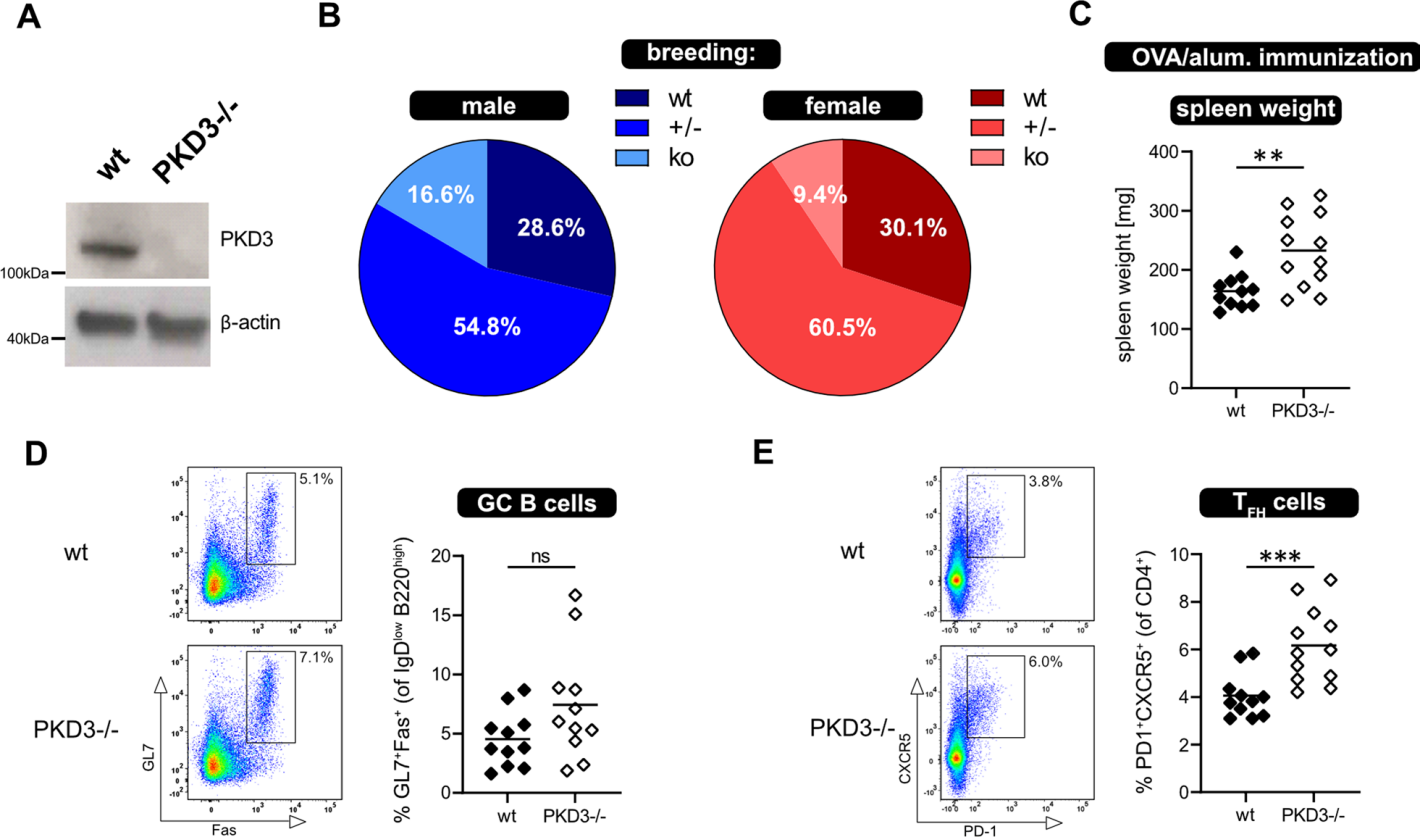
In vivo response to immunization of PKD3-deficient mice. **a** An immunoblot showing that PKD3 protein is absent in PKD3^−/−^ mice. **b** The pie charts illustrate the genotype distribution (wt; ± ; ko) of offspring from PKD3 heterozygous intercross (male in blue, and female in red colors). **c**–**e** The immune response of PKD3^−/−^ and wild type mice was compared 7 days after intraperitoneal immunization with OVA/alum: **c** spleen weight; **d** frequency of splenic GC B cells and **e** Tfh cells, determined ex vivo by flow cytometric analysis. Data are represented as individual values plus mean from 4 independent experiments (n ≥ 11 mice; ns = not significant, **p < 0.01, ***p < 0.001; two-tailed unpaired t-test). Representative FACS dot plots of individual wt and PKD3^−/−^ mice are shown

Based on the rapid and considerable regulation of T cell-expressed PKD isoforms upon stimulation, we suspected a role of PKDs for T cell responses. Since PKD2 had already been implicated in T cell signaling [13, 20], we focused on the little studied PKD3 isoform. Therefore, a PKD3-deficient mouse line, generated by conventional gene targeting in ES cells, was analyzed for their in vivo T cell response to immunization. Analysis of PKD3 protein expression in purified T cells by immunoblot confirmed the absence of PKD3 in PKD3-knockout mice (Fig. 1A), and additionally proved that the anti-PKD3 antibody used for immunoblotting in Fig. 1D specifically recognizes this PKD isoform. We observed that PKD3^−/−^ mice are sterile. Furthermore, heterozygous PKD3 breeding pairs produced PKD3^−/−^ offspring at less than the Mendelian frequency. (Fig. 2B). We performed intraperitoneal OVA/alum immunizations of PKD3-deficient mice and wild type littermates to address the immune response in the absence of PKD3 expression. At day 7 post immunization the mice were euthanized, spleens were weighed and immune cell subsets analyzed by flow cytometry. We observed significantly increased spleen weight in mice lacking PKD3-expression (Fig. 2C). However, while no significant differences in splenic germinal center (GC) B cells were observed (Fig. 2D), the frequency of Tfh cells was significantly increased in the spleens of PKD3-deficient mice (Fig. 2E). These in vivo data suggest that PKD3-deficient mice show enhanced T cell responses to immunization with regard to increased Tfh development.

### PKD3-deficient CD4^+^ T cells show a hyper-responsive phenotype ex vivo, while PKD3 is dispensable for differentiation of naïve T cells in vitro

**Fig. 3:**
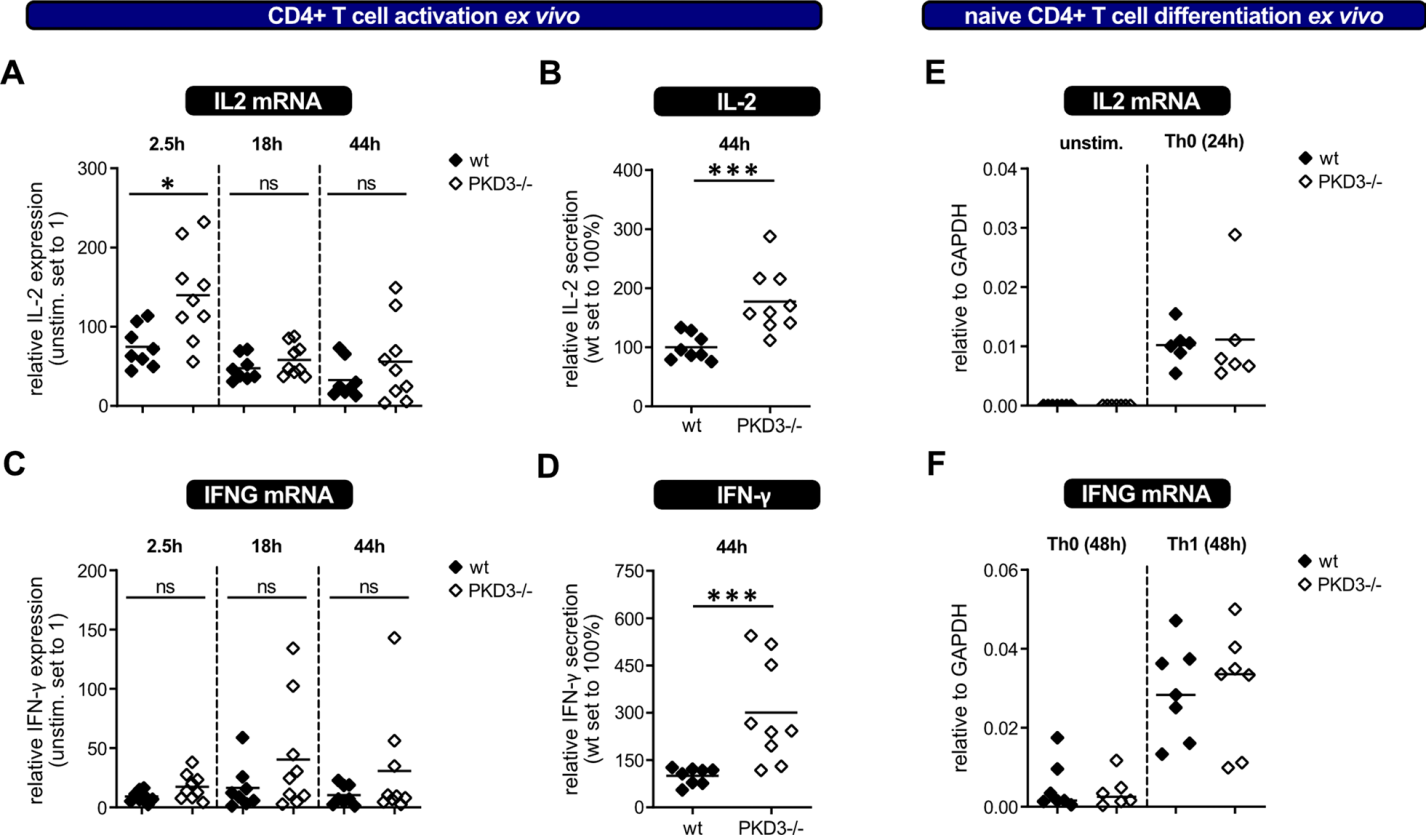
Role of PKD3 for T cell activation in vitro. **a**–**d** CD4^+^ T cells isolated from spleens of either wt or PKD3-deficient mice were stimulated ex vivo with anti-CD3/CD28 antibodies for the indicated time points. IL-2 and IFN-γ expression was assessed either on mRNA level (after 2.5 h, 18 h and 44 h) by qRT-PCR (**a**, **c**) or after 44 h in the culture supernatant by luminex methodology (**b**, **d**). The house keeping gene gapdh was used for normalization of the mRNA data, and the results are depicted relative to unstimulated cells ex vivo. Secreted IL-2 and IFN-γ are shown relative to wt, with the mean wt value of each experiment set to 100. **e**, **f** IL-2 and IFN-γ mRNA expression in naïve sorted CD44^−^CD4^+^ T cells of both genotypes was analyzed by qRT-PCR after sort (unstimulated) and upon in vitro differentiation under non-polarizing (Th0) or Th1-polarizing conditions at the depicted time points. The mRNA data are shown relative to GAPDH. Data are represented as individual values plus mean from 3 independent experiments (n ≥ 8 mice) for a-d and 5 independent experiments (n ≥ 6 mice) for e/f *p < 0.05, ***p < 0.001; two-tailed unpaired t-test followed by Bonferroni’s correction for multiple tests within one graph

Knowing that PKD3-deficient mice mount an enhanced Tfh-response to immunization, we next wanted to analyze the activation response of PKD3-deficient T cells isolated from naïve mice ex vivo. Therefore, CD4^+^ T cells were purified from the spleens of wild type and PKD3^−/−^ littermates and stimulated in vitro with anti-CD3 and anti-CD28 antibodies. PKD3-deficient CD4^+^ T cells expressed more IL-2 mRNA than wild type CD4^+^ T cells early upon T cell stimulation (Fig. 3A). This enhanced IL-2 response was also observed when analyzing secreted IL-2 in the supernatant at day 2 of culture by luminex method (Fig. 3B). We also detected increased IFN-γ in the day 2 supernatant of PKD3^−/−^ CD4^+^ T cells even though this was not significantly different between both genotypes at the mRNA level.

In contrast, when we treated wild type T cells with two distinct pharmacological pan-PKD-inhibitors, proliferation, CD25 up-regulation and IL-2 expression were reduced in a dose-dependent fashion (Additional file 1: Fig. S1).

Given the observed hyper-responsive phenotype of PKD3-deficient CD4^+^ T cells early upon activation ex vivo, we wondered if PKD3 plays any role in the differentiation of naïve CD4^+^ T cells in vitro. To address this question, naive CD44^−^ CD4^+^ T cells were isolated from wild type and PKD3^−/−^ littermates and activated in vitro under Th1-polarizing conditions as well as non-polarizing Th0 conditions. However, there were no significant differences in the IL-2 mRNA levels between freshly sorted naïve CD4^+^ T cells of both genotypes. In addition, similar IL-2 mRNA expression was observed when the naïve T cells isolated from PKD3-deficient mice were stimulated under Th0 conditions (Fig. 3E). Furthermore, IFN-γ mRNA induction in Th1-polarized cells was comparable between wild type and PKD3^−/−^ T cells (Fig. 3F). These experiments suggest that PKD3 is dispensable for activation of naïve CD4^+^ T cells during in vitro culture.

### The peripheral T cell compartment of PKD3-deficient mice is shifted towards an effector/memory phenotype

**Fig. 4:**
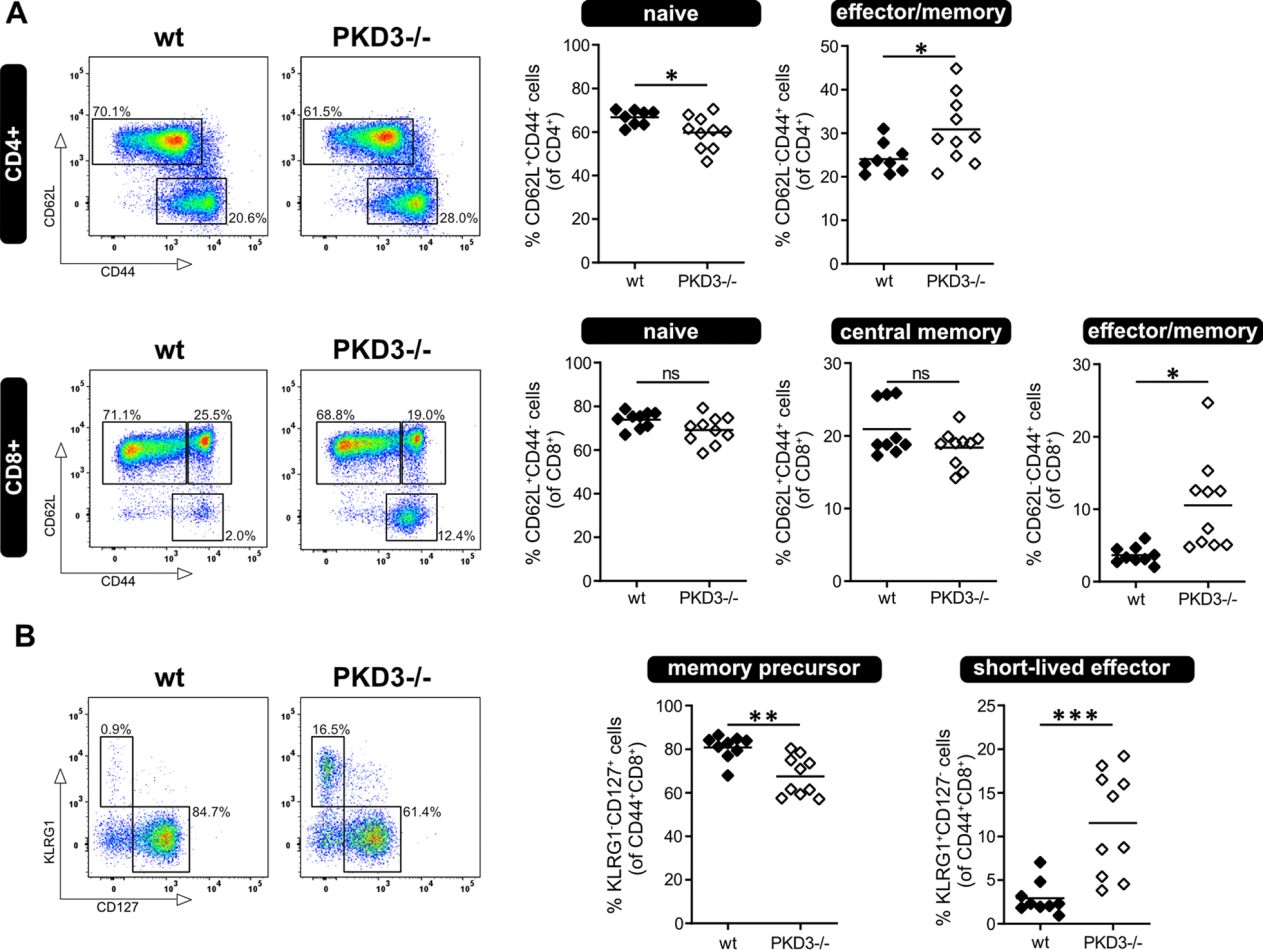
T cells from PKD3-deficient mice show a shift towards an effector/memory phenotype**.** T cell subpopulations from splenocytes of wild type and PKD3^−/−^ mice were analyzed by flow cytometry. **a** Frequencies of naïve and effector/memory T cells were distinguished by CD44 and CD62L staining. Representative FACS dot plots (gated for viable CD4^+^ and CD8^+^ lymphocytes) as well as summarizing graphs are shown for naïve (CD62L^+^CD44^−^) and effector/memory (CD62L^−^CD44^+^) CD4^+^ T cells as well as for naïve (CD62L^+^CD44^−^), central memory (CD62L^+^CD44^+^) and effector/memory (CD62L^−^CD44^+^) CD8^+^ T cells. **b** CD44^+^CD8^+^ T cells are further discriminated into memory precursor (KLRG1^−^CD127^+^) and short-lived effector (KLRG1^+^CD127^−^) cells. Data are represented as individual values plus mean from 4 independent experiments (n ≥ 9 mice; *p < 0.05, **p < 0.01, ***p < 0.001; two-tailed unpaired t-test)

Our results so far point to an enhanced activation response of PKD3-deficient CD4^+^ T cells in vivo as well as ex vivo, while the activation and differentiation of naïve CD4^+^ T cells in vitro is not affected by PKD3-deficiency. To address these seemingly contradictory observations, we performed phenotyping of the splenic T cell compartment of wild type and PKD3^−/−^ littermates at steady state by flow cytometry ex vivo. We observed an increased frequency of effector/memory (CD62L^−^CD44^+^) CD4^+^ T cells while the naïve subset was reduced (Fig. 4A). In addition, within the splenic CD8^+^ T cell compartment of PKD3-deficient mice more cells with an effector/memory phenotype were detected (Fig. 4B). Further analysis of KLRG-1 and CD127 expression on CD8^+^ T cells revealed that PKD3^−/−^ mice have more short-lived effector/memory T cells (T_SLE_, CD44^+^CD127^−^KLRG1^+^; Fig. 4B). Based on these results we conclude that the enhanced activation of PKD3-deficient T cells can be explained by a skewed T cell compartment in vivo.

## Discussion

Up to now, it is not clear whether PKD3 is critical for T cell-mediated immune responses. Taken together, using a PKD3-deficient mouse line, we observed a skewing within the T cell compartment to an increased effector/memory phenotype and furthermore enhanced Tfh-response to OVA/alum immunization. Our study was based on the fact that PKDs are effector kinases downstream of PKCs, which are known to be crucial for TCR signaling. Hence, T cell-intrinsic functions of PKD3 were expected. However, our observations point rather to a T cell-extrinsic role of PKD3, since naïve CD4^+^ T cells either expressing or lacking PKD3 did not show significant differences in vitro. Thus, since we analyzed conventional germline PKD3-knockout mice and this kinase is broadly expressed—also outside the lymphatic compartment—we currently do not know whether this phenotype is conveyed by T cell-intrinsic or -extrinsic functions of PKD3. If our observations are due to a T cell-extrinsic role of PKD3, this is most likely caused by cells interacting with T lymphocytes and thus modulating the T cell compartment. However, little is published regarding an isotype-specific role of PKD3 within the hematopoietic compartment. Moreover, early studies on PKDs could not specify the investigated isoform due to a lack of discriminating antibodies between the highly homologous kinases, used PKD-inhibitors or performed ectopic expression in cell lines. Therefore, it is difficult to draw conclusions on PKD isoform-specific functions.

Nevertheless, one publication showed that human primary B lymphocytes are similar to T lymphocytes in that they express only PKD2 and PKD3 [21]. Additionally, also in B cells PKD phosphorylation is linked to BCR signaling [21–23]. However, it is currently not clear if PKD2 and PKD3 have redundant or distinct functions in B cells. In addition, there are some implications of PKDs in the innate immune cell compartment. Thus, in peritoneal macrophages conditional knockout of PKD1 along with PKD3 impaired NLRP3 inflammasome activation; yet this suppression was more pronounced when a PKD inhibitor was used, suggesting that all three PKD isoforms can contribute to activation of the NLRP3 inflammasome [24]. Recently, PKD3-specific functions for macrophage activation have been shown using independent PKD3-deficient mouse lines (conventional as well as conditional myeloid-cell-specific). The authors of this study observed skewing towards an alternative activated phenotype and thereby an inclination to spontaneous liver fibrosis after four months when PKD3 was absent [25]. In contrast to these observations, we have not seen any macroscopic abnormalities when performing necropsy of our PKD3-deficient mouse line up to the age of 16 months (data not shown). Additionally, although this study did not focus on the adaptive immune system, their PKD3^−/−^ mice had enlarged spleens, pointing to an enhanced immune activation status.

Currently, to the best of our knowledge, no reports exist on the role of PKD3 for dendritic cell or thymic stromal cell function; two cell types, which contribute the most to T cell homeostasis in vivo for example by providing cytokines such as IL-15 and stimulation via MHC molecules and thus, regulate the distinct niches within the T cell compartment [26]. However, in a broader sense, all MHC class I expressing cells can interact with and thus modulate T cells, as during viral infections or cancer. Especially in the latter case, PKD3 seems to play non-redundant roles in tumor cells. Hence, several studies have shown that this isoform is upregulated in human cancer types such as triple-negative breast cancer [27, 28], gastric cancer [29] and prostate cancer [30, 31], which correlates with both the pathological grade as well as metastatic progression. Via overexpression or siRNA-mediated knockdown in cancer cell lines, it was shown that PKD3 is a positive regulator of proliferation, viability and migration. Furthermore, PKD3-knockout did slow down tumor growth in subcutaneous tumor xenografts [28–30]. Interestingly, tumor-intrinsic PKD3 has been described to promote immune escape by enhancing expression of Fas and PD-L1 as well as secretion of TGF-β, CCL-21 and IL-10, all of which are critical to establish an immunosuppressive tumor microenvironment [32, 33]. Indeed, enhanced activation and proliferation of human T cells was observed when PBMCs were co-cultured with PKD3 siRNA-transfected tumor cells [33]. Hence, at least in this context, T cell function seems to be regulated also by T cell-extrinsic PKD3.

On the contrary, the proposed involvement of PKDs in TCR signaling, especially its dominant regulation upon T cell activation as well as our results obtained with PKD inhibitor-treated T cells in vitro suggest rather a T cell-intrinsic role of PKD3. However, both inhibitors that were used in the current study are pan-PKD-inhibitors; therefore, we cannot distinguish between effects mediated by suppression of PKD2 and of PKD3. Of note, PKCθ-deficient T cells show a similar phenotype as PKD inhibitor-treated T cells upon in vitro stimulation with anti-CD3 and anti-CD28 antibodies [1, 3]. Which raises questions concerning the specificity of the small molecule inhibitors, which have been used in several publication as PKD-specific inhibitors [24, 34, 35]. However, to the best of our knowledge, no data on the treatment of primary T lymphocytes with PKD-inhibitors have been published so far. Nevertheless, if PKDs are downstream of novel PKCs it is not surprising that PKD-inhibitor treatment leads to a phenotype that resembles PKCθ^−/−^ T cells. Of note, we observed that once T cells have been stimulated and hence, have down-regulated PKD2 and PKD3, they are not sensitive anymore to the PKD inhibitor (data not shown), indicating that the used inhibitor is indeed quite specific. In addition, this might suggest that PKDs are only important very early upon activation. Once activation has taken place and they are downregulated, T cell activation is independent of PKDs.

A major question, which has arisen from our data obtained from investigation of the T cell compartment of PKD3-deficient mice (especially when claiming a PKD3 T cell-intrinsic effect) was, whether the two highly homologous isoforms, PKD2 and PKD3, carry out similar or opposing roles.

Next to the highly conserved structure, the assumption of similar functions is particularly supported by a recent publication identifying PKD2 as a critical kinase in a forward genetic screen using aberrant IgE levels in response to immunization with OVA/alum as a readout [17]. The authors could elucidate that these effects are secondary to an increased transition from naïve CD4^+^ to Tfh cells when functional PKD2 is absent. In line with this, in an earlier study, using different TCR-transgenic mice deficient for PKD2, significantly bigger spleens were observed and a role for PKD2 as a negative regulator of T cell activation was suggested [20]. These findings resemble our results of an enhanced T cell response to OVA/alum immunization. In this respect, Ishikawa et al*.* proposed not only a complementary, but even redundant function of the two PKD isoforms for T cell development [18]. Using Lck-Cre mediated ablation of either PKD2, PKD3 or both, they revealed a reduction of CD4^+^ SP thymocytes only in the simultaneous absence of both PKD isoforms. Thus, when being redundant during development, this presupposes similar functions, which may be also true for mature T cells, especially when considering that PKDs are involved directly in TCR signaling. In addition, there are several studies showing redundant functions between the individual PKD isoforms. Hence, in the chicken B cell line DT40 two PKD isoforms (resembling mammalian PKD1 and PKD3) both phosphorylate and regulate histone deacetylases (HDAC) 5 and HDAC7 [36]. Beyond this, there are reported redundancies between PKD isoforms involved in golgi organization and protein transport, NF-κB activation, cell survival responses and chemokine release from Toll-like receptor activated epithelial cells [37–44].

On the other hand, our data show partially divergent effects of PKD3 ablation to the ones described with PKD2-knockout mice. In contrast to our findings showing that a loss of PKD3 leads to increased IL-2 as wells as IFN-γ production ex vivo, other groups have observed that the amount of PKD2 in T cells positively correlates with their amount of IFN-γ secretion [16]. In line with this, an earlier study showed that mice lacking PKD2 catalytic activity mount an impaired T cell-dependent antibody response [13]. Interestingly, besides observing PKD2’s digital activation behavior upon T cell activation, Navarro et al*.* have concluded that this isoform serves as a regulator of the transition from naïve into the effector T cell pool, depending on the strength of the TCR signal. [16]. In accordance with this, adoptive transfer of OT-I T cells expressing a kinase-dead mutant of PKD2 and subsequent immunization with the corresponding antigen SIINFEKL together with LPS, results in significantly less KLRG1^+^ effector T cells. Thus, it is conceivable that while a loss of PKD2 impedes the transition to effector cells this process is favored by the loss of PKD3. However, the composition of the splenic T cell compartment of PKD2-deficient mice has not been published so far.

Taken together, although it seems paradox, our observations with PKD3-deficient mice partially recapitulate and partially contradict the results of PKD2-knockout studies. Nevertheless, and despite high homology between the PKD isoforms, this is reflected also within other studies of PKD isoforms, such as work published by Marklund et al. Using a model system, where pre-T cells expressed a constitutively active kinase domain of PKD1, this group has surprisingly observed that depending on the developmental stage, PKD1’s catalytic domain either negatively (DN4) or positively (DN3) regulated TCR signaling. Thus, the authors concluded that PKD can act either as a positive or a negative regulator of T cells, depending on cell context [45].

Conversely, it is also clear that many functions of PKDs are unique to one of two of the isoforms. Thus, for instance, phosphorylation of phosphoinositide 4-kinase IIIβ is regulated by PKD1 and PKD2, but not PKD3 [46]. Moreover, differently timed as well as cell type-specific expression patterns of PKD isoforms point rather to non-redundant PKD functions.

Thus, from the data so far, we cannot simply conclude if similar, redundant or even opposing roles exist between PKD2 and PKD3. As previous studies have suggested, the function of PKD isoforms likely depends on the cellular context and additionally maybe on the activation level and differentiation status. To address this question and in addition the issue of intrinsic versus extrinsic roles of PKD3 for the T cell compartment, a T cell-specific knockout or knockin system (e.g. using Cre driven by the CD4 promoter) would be required, preferentially targeting either PKD2 or PKD3 or both, if feasible best in an inducible manner to also address different developmental stages. Although interesting, this goes beyond the scope of the current study.

## Conclusion

The present study analyzes for the first time the peripheral T cell compartment of a germline PKD3-knockout mouse line. We observed that PKD3-deficiency increases the proportion of cells with an effector/memory phenotype among CD4^+^ as well as CD8^+^ T cells. Hence, PKD3^−/−^ mice showed an enhanced T cell response to immunization*,* and increased T cell activation upon TCR stimulation ex vivo, which however was not observed with purified naïve T cells. These seemingly contradictory observations might be explained by the skewed T cell compartment of PKD3-deficient mice already under steady state conditions. These observations, together with our results of strong regulation of PKD3 expression in wild type T cells upon TCR trigger, suggest that this kinase acts as a modulator of T cell homeostasis. Nonetheless, we currently cannot answer the question whether this is due to T cell-intrisic or -extrinsic mechanisms. Yet the present study intends to serve as a starting point on the way to elucidate PKD3-specific functions in the murine T cell compartment.

## Supplementary Information

**Additional file 1**: **Figure S1**. Response of wild type T cells to PKD inhibitors in vitro. **a, b** MACS-sorted CD4+ T cells were treated with several concentrations of either one of two low molecular weight inhibitors (CRT0066101 in orange or CID2011756 in blue) one hour prior to stimulation with anti-CD3/CD28 antibodies. On day 2 of culture CD25 and Ki-67 expression were analyzed by flow cytometry to assess activation and proliferation, respectively. **c** IL-2 expression upon 4-hour stimulation of splenocytes with phorbol ester/ionomycin in the presence of brefeldin A and the depicted concentration of CRT was analyzed by intracellular staining and flow cytometry. Representative FACS histograms showing IL-2 (gated on CD4+ T cells) and summarizing graphs are shown. ≥ 2 independent experiments; n ≥ 4 mice analyzed individually.

## Data Availability

All data used in this study are available from the corresponding author on reasonable requests.

## Abbreviations

PKC: Protein kinase C
PKD: Protein kinase D
IL-2: Interleukin-2
TCR: T cell receptor, NF-κB: nuclear factor κ B
wt: Wild type
ko: Knockout
