# Supplementary material for: Addressing the role of PKD3 in the T cell compartment with knockout mice
Source: Cell Commun Signal. 2022 Apr 19;20:54. doi: 10.1186/s12964-022-00864-w (PMC9020081; doi:10.1186/s12964-022-00864-w)
Supplement: Supplementary file 2 — Additional file 1: Figure S1. Response of wild type T cells to PKD inhibitors in vitro. a, b MACS-sorted CD4+ T cells were treated with several concentrations of either one of two low molecular weight inhibitors (CRT0066101 in orange or CID2011756 in blue) one hour prior to stimulation with anti-CD3/CD28 antibodies. On day 2 of culture CD25 and Ki-67 expression were analyzed by flow cytometry to assess activation and proliferation, respectively. c IL-2 expression upon 4-hour stimulation of splenocytes with phorbol ester/ionomycin in the presence of brefeldin A and the depicted concentration of CRT was analyzed by intracellular staining and flow cytometry. Representative FACS histograms showing IL-2 (gated on CD4+ T cells) and summarizing graphs are shown. ≥ 2 independent experiments; n ≥ 4 mice analyzed individually. [file 12964_2022_864_MOESM2_ESM.pdf]

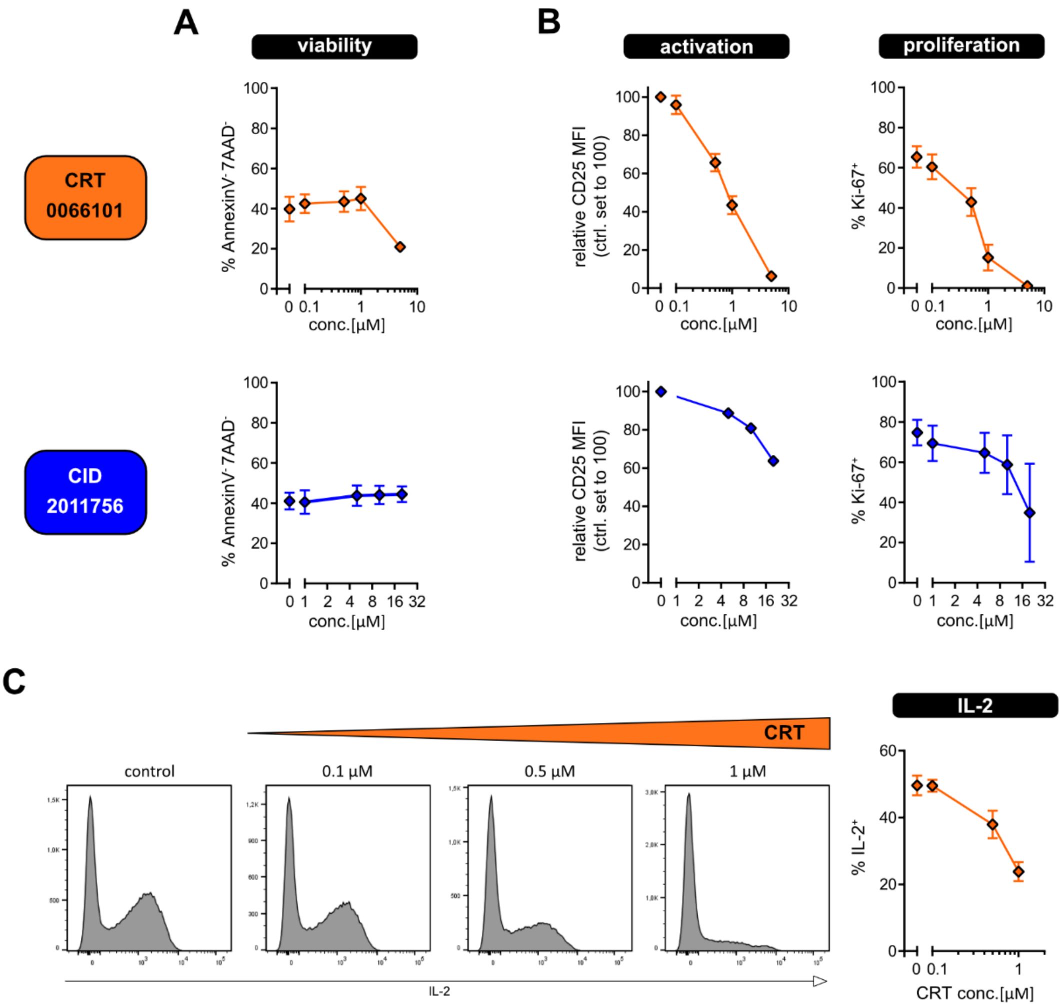


**Suppl. Fig. 1** Response of wild type T cells to PKD inhibitors *in vitro*. **a, b** MACS-sorted $CD4^+$ T cells were treated with several concentrations of either one of two low molecular weight inhibitors (CRT0066101 in orange or CID2011756 in blue) one hour prior to stimulation with anti-CD3/CD28 antibodies. On day 2 of culture CD25 and Ki-67 expression were analyzed by flow cytometry to assess activation and proliferation, respectively. **c** IL-2 expression upon 4-hour stimulation of splenocytes with phorbol ester/ionomycin in the presence of brefeldin A and the depicted concentration of CRT was analyzed by intracellular staining and flow cytometry. Representative FACS histograms showing IL-2 (gated on $CD4^+$ T cells) and summarizing graphs are shown. ≥ 2 independent experiments; n ≥ 4 mice analyzed individually
